# EXPLORING THE RELATIONSHIP BETWEEN MULTIDOMAINS OF FRAILTY AND MORTALITY IN OLDER ADULTS LIVING IN COMMUNITY

**DOI:** 10.1093/geroni/igae098.2759

**Published:** 2024-12-31

**Authors:** Yuelin Li, Rhayun Song, Jisu Seo

**Affiliations:** Chungnam National University, Daejeon, Republic of Korea; Chungnam National University, Daejeon, Republic of Korea; Chungnam National University, Daejeon, Republic of Korea

## Abstract

The global population’s aging presents health challenges. This study aimed to explore the relationship between various domains of frailty (physical, cognitive, social, and multidimensional) on all-cause mortality in community-dwelling older adults. Studies were searched in five databases up to December 5, 2023. Quality and Risk of Bias assessment were assessed using the ROBINS-E tool. A meta-analysis was performed to synthesize pooled mortality risk estimates by using the random-effects model. A total of 53 studies were included for systematic review, with 49 eligible for meta-analysis, involving 153,237 participants and over 31,021 deaths. The quality assessment revealed that 10 were low risk (18.9%), 7 had some concerns (13.2%), 34 were high risk (64.2%), and 2 were considered very high risk (3.7%). Results indicated that frail older adults had a significantly higher mortality risk than robust ones, consistent across frailty domains (physical HR=1.72, 95 CI%:1.50-1.97; social HR=1.17, 95 CI%:1.04-1.31; multidimensional HR=2.37, 95 CI%: 2.10-2.68; cognitive HR=1.48, 95 CI%:1.09-2.01). Our findings highlight that various domains of frailty are strongly and robustly associated with all-cause mortality in older adults. This relationship between multidimensional frailty and mortality is stronger than that of a single type of frailty. Therefore, although focusing on a single frailty can improve the well-being of the older population, interventions targeting multiple aspects of frailty are more effective in improving the quality of life and longevity of this population.

